# Rinsing sampling of core needle biopsy for flow cytometric analysis: A favorable method for lymphoma diagnosis

**DOI:** 10.1002/cam4.3540

**Published:** 2020-10-17

**Authors:** Pei‐Dong Chi, Yi‐Jun Liu, Yu‐Hua Huang, Ming‐Jie Mao, Yu Wang, Zhi‐Ming Li, Jian Li

**Affiliations:** ^1^ Sun Yat‐sen University Cancer Center State Key Laboratory of Oncology in South China Collaborative Innovation Center for Cancer Medicine Guangzhou Guangdong P.R. China; ^2^ Department of Clinical Laboratory Sun Yat‐Sen University Cancer Center Guangzhou Guangdong P.R. China; ^3^ Department of Pathology Sun Yat‐Sen University Cancer Center Guangzhou Guangdong P.R. China; ^4^ Department of Internal Medicine Sun Yat‐Sen University Cancer Center Guangzhou Guangdong P.R. China; ^5^ Department of Diagnostic and Interventional Ultrasound Sun Yat‐Sen University Cancer Center Guangzhou Guangdong P.R. China

**Keywords:** core needle biopsy, fine needle aspiration, flow cytometry, lymphoma, pathologic diagnosis

## Abstract

**Background:**

Conventional protocols utilize core needle biopsy (CNB) or fine needle aspiration (FNA) to produce cell suspension for flow cytometry (FCM) is a diagnostic challenge for lymphoid malignancies. We aim to develop an alternative CNB rinsing technique (RT) to produce cell suspension for FCM during this mini‐invasive procedure of CNB for lymphoma diagnosis.

**Methods:**

FNA and CNB specimens from the same lesion of 93 patients with suspected lymphoma were collected under the guidance of B‐ultrasound simultaneously. The fresh CNB samples were prepared to cell suspension by RT for FCM immunophenotyping analysis (Group CNB‐RT). Then, the CNB tissues after performing the RT process and the fresh FNA tissues were processed by conventional tissue cell suspension (TCS) technique to obtain the cell suspensions (Groups of CNB‐TCS & FNA‐TCS), respectively, as comparison. The diagnostic efficacies, as well as the concordances of the FCM results with reference to the morphologic diagnoses were compared in these three groups.

**Results:**

RT could yield sufficient cells for FCM immunophenotyping analysis, though a lower cell numbers compared to TCS technique. The diagnostic concordance was comparable in group CNB‐RT (91.1%) to the group CNB‐TCS (88.9%) and group FNA‐TCS (88.4%) (*p* = 0.819). The diagnostic sensitivity and specificity of CNB‐RT (91.1%; 100%) was not inferior to that of CNB‐TCS (88.9%; 100%) and FNA‐TCS (88.4%; 98.8%).

**Conclusions:**

This study shows the CNB‐RT presented non‐inferior diagnostic concordance and efficacy as compared to the TCS technique. CNB‐RT has the potential to produce cell suspension for FCM immunophenotyping while preserving tissue for lymphoma diagnosis and research.

## Graphical abstract

In this study, we developed an alternative core needle biopsy (CNB) rinsing technique (RT) to produce cell suspension for flow cytometry (FCM) during this mini‐invasive procedure of CNB for lymphoma diagnosis. We found that the CNB‐RT presented non‐inferior diagnostic concordance and efficacy as compared to the conventional tissue cell suspension (TCS) technique. CNB‐RT has the potential to produce cell suspension for FCM immunophenotyping while preserving tissue for lymphoma diagnosis and research.

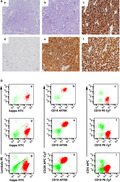

## 1 INTRODUCTION

The specific lymphoma subtype is difficult due to the inherent complexity of the past and current lymphoma classifications.^1^ The defining criteria for these diseases as well as the appropriate therapeutic strategies are mainly based on histologic findings from surgically excised specimens,^2^, ^3^, ^4^ in which the histologic features are essential in the diagnosis of lymphoma, or the histologic patterns, such as diffuse, nodular, and mixed nodular and diffuse, were crucial for the classification of lymphoma.^5^, ^6^, ^7^ Nevertheless, recent years have seen an increasing reliance on core needle biopsy (CNB) and fine needle aspiration (FNA) to evaluate lymphadenopathy, although lacking of histologic patterns are the disadvantages of FNA and most CNB.^8^, ^9^, ^10^ The choice of pathologists and oncologists with this shift from excisional biopsies to FNA/CNB has been facilitated by the advent of ancillary technique, such as flow cytometry (FCM), karyotypic analysis, and molecular diagnostic techniques. Numerous publications have reported the advances in using these ancillary studies for lymphoma diagnosis according to the WHO subclassification criterion.^11^, ^12^, ^13^, ^14^, ^15^, ^16^, ^17^ In many institutes, FNA/CNB has become the primary diagnostic procedure for patients with suspected lymphoma.^13^ Lots of studies demonstrate that FNA and CNB with FCM represent a viable alternative in the diagnosis and subclassification of primary and recurrent lymphoma, as long as the number and size of cores for morphologic studies are not compromised.^8^, ^9^, ^10^, ^11^, ^13^, ^18^, ^19^, ^20^ However, almost all the cell suspensions used for the FCM immunophenotyping analysis in those published literatures were prepared by the conventional tissue cell suspension (TCS). In this mini‐invasive procedure, the FNA and CNB specimens with little tissue, which were originally used for morphological examination, were inevitably split for FCM detection. And the remaining tissue may be insufficient in quantity or quality for an accurate and definitive histologic diagnosis.^21^

Previous literatures focused on the discussion of the role of FCM in the diagnosis of lymphoma rather than the improvement of cell suspension preparation method. It has been reported that bone marrow core biopsy specimens were prepared to cell suspension by "vortex" method for FCM immunophenotyping detection.^22^, ^23^ However, it is rare to report the cell suspension preparation method using CNB specimen, rinsing technique (RT), as we described here. There was only one retrospective article about RT using CNB specimen to prepare the cell suspension for FCM study, in which un‐paired data and un‐equal number of cases in both RT and TCS groups were the main limitations.^21^ Herein, we designed a prospective parallel study to evaluate the CNB rinsing technique in the function of FCM diagnosis for lymphoid malignancies, in which conventional TCS technique using CNB and FNA tissues were compared. Meanwhile, the diagnostic efficacies, as well as the concordances of the FCM results with reference to the morphologic diagnoses were compared in these three groups. Our aims are to investigate the diagnostic performance of CNB‐RT for FCM analysis compared to the conventional TCS technique, and then, to evaluate whether this new method of preparing cell suspension could be applied in the primary diagnostic strategy of patients with suspected lymphoma by the mini‐invasive approach of CNB.

## 2 MATERIALS AND METHODS

### 2.1 Patients and study design

About 93 consecutive patients with suspected lymphoma were recruited in this prospective study from May 3, 2017 to February 8, 2018. We collected the FNA and CNB specimens from the same lesion simultaneously during Ultrasound guided CNB for these patients. Each patient was numbered from case 1 to case 93 according to the sampling sequence. The lesion sites were classified into superficial versus deep‐seated, and lymph node versus extranodal lesions. The superficial lesions included the cervical, clavicular region, parotid region, lateral lobe of thyroid, axilla, breast, groin, thigh muscle layer, and testis. The deep‐seated lesions included the thorax, abdomen, retroperitoneum, and pelvis regions.

The primarily collected CNB tissues (at least two cores) were sent to pathological department for the histopathological evaluation which was regarded as the standard reference. Additional one core of CNB tissue was subsequently collected for preparation of the cell suspension by RT (Group CNB‐RT), which was processed as follows: One core of fresh CNB was submerged and stirred gently for three to four times in phosphate buffer (PBS) with PH7.4 using sterile forceps at room temperature immediately after they were collected. The cell suspension in PBS medium which was obtained by RT, devoid of tissue, was sent for FCM immunophenotyping analysis immediately. Then, the CNB tissues after performing the RT process and the fresh FNA tissues were processed by TCS to obtain the cell suspensions for FCM detection respectively (Groups of CNB‐TCS and FNA‐TCS). The conventional TCS technique was performed using the traditional protocol in which a single or a portion of representative tissue core was disaggregated mechanically with knives and needles to produce a cell suspension for FCM analysis.^21^ Consequently, we obtained a total of 279 cell suspension samples prepared for FCM immunophenotyping detection, 93 samples for each group.

The diagnostic concordances between the FCM immunophenotyping results and the morphologic diagnoses as well as the diagnostic efficacy of each group were compared in these three groups of consistently matched data. The FCM data of all patients in the study were analyzed by the same pathologist（Dr. Chi) without blinding to the technique type. The pathologic reports of CNB specimen biopsies were issued by the others pathologists on duty. It was double blind for that the pathologists of FCM immunophenotyping and biopsy detection did not know each other's results before the reports were issued. Dr. Chi judged whether the results of FCM immunophenotyping and CNB biopsy were concordant according to the following rules first. Then, a review group composed of Dr. Chi, Dr. Huang and Prof. ZM. Li would review all the reports of FCM and biopsies of each patient, and make a final judgment on the concordance of the results. For those discordant cases, Dr. Huang was responsible for reanalyzing the FCM data and rereading the biopsy slides. If the analysis results of Dr. Huang are consistent with initial reports, respectively, the case would still be classified as non‐concordance. If not, two or more other pathologists would be recruited to reanalyze the FCM data and CNB biopsy. The consensus diagnoses of the discordant cases were made by these pathologists. The concordant judgment standard of each disease entity was as follows: For cases of B‐cell and T‐cell lymphoma which were diagnosed by morphology, concordances were made if FCM analysis detected a monotypic B‐cell population with a restricted light chain expression or a dominant T‐cell population with aberrant phenotypes. Negative results or misdiagnosis to the other diseases by FCM would be considered discordant. For reactive and atypical cases, concordances were considered if the FCM results were negative, whereas discordances were made if diagnosed as lymphoma by FCM. Concordant judgments would be made if a borderline light‐chain excess or a small subset of monotypic B‐cells or abnormal T ‐cells were detected by FCM in suspicious cases diagnosed by morphology, otherwise it was considered discordant. Finally, for classical Hodgkin lymphoma (CHL) and non‐hematopoietic system diseases, a negative FCM finding for these two disease categories was considered concordant to morphologic diagnosis. Because the panels with B‐ and T‐cell markers we used for FCM analyses usually were limited without CD30, CD15, or other specific markers for CHL and non‐hematopoietic system diseases since the insufficient cells; Furthermore, FCM did not play an important role in the diagnosis of CHL and non‐hematopoietic system diseases due to the lack of a clonal population or specific antibodies. In our study, a total of 28 FCM specimens from three groups of CNB‐RT, CNB‐TCS, and FNA‐TCS were not concordant with the biopsy results, which were from 16 CNB tissue specimens. After reanalyzing by Dr. Huang, all these 28 FCM specimens and 16 CNB biopsy specimens were consistent with initial reports respectively and were still classified to the discordant cases. Thus, No more pathologists were arranged to review the data and slides.

Sensitivity and specificity were used to evaluate the diagnostic efficacies of these three cell suspension methods. According to the definition of sensitivity (true positive rate), the concordant rate between FCM immunophenotyping and biopsy is consistent with the value of sensitivity.^24^ We also calculated the specificities (true negative rate) of three cell suspension preparation methods.

Prior to the use of the tissues, written informed consent was obtained from each of the patients. This study was approved by the ethics committees of Sun Yat‐sen University Cancer Center (SYSUCC, Guangdong, China) (approval number GZR2014‐044) and was conducted in accordance with the ethical standards of the World Medical Association Declaration of Helsinki. The authenticity of this article had been validated by uploading the key raw data onto the Research Data Deposit public platform (www. researchdata.org.cn), with the approval RDD number as RDDA2020001528.

### 2.2 Morphological examination

The cases of CNB with adequate material were evaluated by hematopathologists based on the WHO classification of tumors of hematopoietic and lymphoid tissues 2016.^1^ The specimen was designated as insufficient for morphologic evaluation if it was too small or unreadable due to extensive necrosis, severe crush or fixation artifact. An excisional biopsy was recommended if a specific diagnosis could not be made.

### 2.3 Flow cytometric detection

Before the FCM detection, an accurate dilution of cells in trypan blue vital stain counted on a hemacytometer chamber which gave an accurate volume allowed assessment of the viability as well as the number of cells in each suspension derived from three groups of CNB‐RT, CNB‐TCS, and FNA‐TCS.^25^ Then, the suspensions with sufficient viable cells were analyzed by FCM. Eight‐color FCM analysis was performed on FACScantoⅡ flow cytometer (BD Biosciences, San Jose, CA) according to the standard procedures,^26^ and data were analyzed using the FACSDiva software (BD Biosciences, San Jose, CA). Because we did not know in advance which type of lymphoma the patient had, thus, both B‐cell tube (including CD45, CD19, CD20, CD5, CD10, CD38, and surface immunoglobulin light chains (kappa and lambda)) and T/NK‐cell tube (including CD45, CD3, CD4, CD8, CD2, CD7, CD5, and CD56) were performed to each cell suspension. A diagnosis of B‐cell non‐Hodgkin lymphoma (B‐NHL) was made in the presence of a monotypic B‐cell population with a restricted light chain expression and/or aberrant antigen expression. T‐cell lymphoproliferative disorder was diagnosed if there was a dominant T‐cell population with aberrant phenotypes such as lacking and/or abnormal distribution of one or more T‐cell antigens. A case was considered suspicious for lymphoproliferative disorder if analysis detected a borderline light‐chain excess or a small subset of monotypic B‐cells or abnormal T‐cells.

### 2.4 Statistical analysis

All statistical analyses were performed using SPSS 19.0 statistical software package (SPSS Inc., Chicago, IL). The comparison of the cell number was analyzed by Mann–Whitney *U* Test. Fisher's exact test was used to analyze the difference in diagnostic concordance among three groups. The data with a normal distribution were expressed as mean ±standard deviation, while the data with nonnormal distribution was expressed as median (range). All tests were two‐tailed, and *p* value <0.05 was considered significant.

## 3 RESULTS

### 3.1 Patient demographic and clinical data

A total of 93 patients with 279 cell suspension samples collected from FNA and CNB specimens in the same lesion simultaneously were enrolled. The mean age of the patients ± the standard deviation was 49.1 ± 17.2 years (range, 18‐94 years) and included 50 men and 43 women. There were 49 cases of superficial lesion and 44 cases of deep lesion. The nodal and extranodal lesions were 45 and 48 cases, respectively.

A total of 13 cases yielded an insufficient cell number for FCM within the cohorts. Both 3 cases (3/93; 3.2%) in groups CNB‐RT and CNB‐TCS, respectively; seven cases (7/93; 7.5%) in group FNA‐TCS. FCM was performed on the rest of quantity sufficient cases, including both 90 cases (90/93; 96.8%) in the groups CNB‐RT and CNB‐TCS, respectively; 86 cases (86/93; 92.5%) in the group FNA‐TCS. No significant difference was found in the cases of insufficient cell number for FCM analysis among these three groups (*p* = 0.094) (Table 1). Quantity not sufficient for FCM detection was abbreviated as QNS. The sequence numbers of QNS cases in each group were listed according to the disease classification (Table S1). The viable cell numbers of groups CNB‐RT, CNB‐TCS, and FNA‐TCS for FCM analysis were 0.232 × 10^6^ (0.002‐2.080), 1.060 × 10^6^ (0.004‐6.338), and 3.296 × 10^6^ (0.003‐59.803), respectively. Group FNA‐TCS had more cell number than the other two groups (*p* < 0.001).

**Table 1:** Quantity control for FCM analysis and comparison of the diagnostic concordance between morphology and FCM in quantity sufficient cases in three cell suspension preparation methods

Result	CNB‐RT^a^	CNB‐TCS^b^	FNA‐TCS^c^	Total	*p*‐value
	No. (%)	No. (%)	No. (%)		
Quantity not sufficient	3 (3.2)	3 (3.2)	7 (7.5)	13	0.094
Quantity sufficient					
Concordant	82 (91.1)	80 (88.9)	76 (88.4)	238	0.819
Discordant	8 (8.9)	10 (11.1)	10 (11.6)	28	
Total	93	93	93	279	

^a^ CNB‐RT, core needle biopsy‐rinsing technique.

^b^ CNB‐TCS, core needle biopsy‐tissue cell suspension

^c^ FNA‐TCS, fine needle aspiration‐tissue cell suspension.

### 3.2 Diagnostic concordance of the three cell suspension preparation methods

The concordant and discordant cases with sufficient cells for FCM detection in three groups are listed in Table 1. We found that group CNB‐RT had a slightly higher concordant rate (82/90; 91.1%) than groups CNB‐TCS (80/90; 88.9%) and FNA‐TCS (76/86; 88.4%) though there was no significant difference among three groups (*p* = 0.819). More specifically, the concordant cases between the FCM results and morphologic diagnosis in three groups according to the categories of morphological diagnosis are listed in Table 2. We classified the morphological diagnosis to eight categories. The concordant rate reached 100% in low‐grade B‐cell lymphomas in both groups CNB‐RT (15 samples) and CNB‐TCS (15 samples). One of fifteen samples (6.7%) of low‐grade B‐cell lymphomas in group FNA‐TCS was negative of FCM result. Therefore, the concordant rate of which was 93.3% (14/15). The 15 cases of low‐grade B‐cell lymphomas include three chronic lymphocytic leukemia/small B‐cell lymphocytic lymphoma (CLL/SLL), one mantle cell lymphoma (MCL), four marginal zone lymphoma (MZL), and seven follicular lymphoma (FL).

**Table 2:** Comparison of the diagnostic efficacy in three cell suspension preparation methods for FCM analysis with reference to morphological diagnosis

Morphological Diagnosis^d^	CNB‐RT^a^				CNB‐TCS^b^				FNA‐TCS^c^			
	No.	Concordant No. (%)	Se^e^ (%)	Sp^f^ (%)	No.	Concordant No. (%)	Se (%)	Sp (%)	No.	Concordant No. (%)	Se (%)	Sp (%)
Low grade B‐cell lymphoma^g^	15	15 (100)	100	100	15	15 (100)	100	100	15	14 (93.3)	93.3	100
Diffuse large B‐cell lymphoma	26	22 (84.6)	84.6	100	26	20 (76.9)	76.9	100	26	21 (80.8)	80.8	100
B‐UCL^h^	2	0	0	100	2	0	0	100	2	0	0	100
T‐cell lymphoma	3	1 (33.3)	33.3	100	3	1 (33.3)	33.3	100	2	1 (50.0)	50	98.8
CHL^i^	7	7 (100)	100	100	7	7 (100)	100	100	7	7 (100)	100	100
Reactive^j^	10	10 (100)	100	100	10	10 (100)	100	100	9	9 (100)	100	100
Atypical/Suspicious^k^	7	7 (100)	100	100	7	7 (100)	100	100	7	7 (100)	100	100
Non‐hematopoietic system diseases^l^	20	20 (100)	100	100	20	20 (100)	100	100	18	17 (94.4)	94.4	100
Total	90	82 (91.1)	91.1	100	90	80 (88.9)	88.9	100	86	76 (88.4)	88.4	98.8

^a^ CNB‐RT, core needle biopsy‐rinsing technique.

^b^ CNB‐TCS, core needle biopsy‐tissue cell suspension.

^c^ FNA‐TCS, fine needle aspiration‐tissue cell suspension.

^d^ Diagnosis category referred to the overall final histologic diagnosis.

^e^ Se, sensitivity.

^f^ Sp, specificity.

^g^ Low grade B‐cell Lymphoma included chronic lymphocytic leukemia/small B‐cell lymphocytic lymphoma (CLL/SLL), mantle cell lymphoma (MCL), marginal zone lymphoma (MZL), and follicular lymphoma (FL).

^h^ B‐UCL, unclassified B‐cell lymphoma.

^i^ CHL, classical Hodgkin lymphoma.

^j^ Reactive included the negative or granulomatous lymphadenitis cases diagnosed by histology.

^k^ Atypical/suspicious included the cases diagnosed as reactive hyperplasia (RH), atypical lymphoid proliferation (ALP), or was suspicious for lymphoma by histology.

^l^ Non‐hematopoietic system diseases included gastrointestinal stromal tumor, lymphoepithelioma‐like carcinoma, neuroendocrine neoplasm, low‐differentiated adenocarcinoma, low‐differentiated squamous cell carcinoma, thymoma, small cell carcinoma, fibroma, lympho papillary cystadenoma, and neurinoma.

For classical Hodgkin lymphoma (CHL), reactive and atypical /suspicious lymphadenopathies, FCM and morphologic evaluation also showed 100% concordance rate among three groups, respectively.

Twenty cases (58 samples) of non‐hematopoietic system diseases were detected by FCM including one case of gastrointestinal stromal tumor, low‐differentiated squamous cell carcinoma, fibroma, lympho papillary cystadenoma, and neurinoma, respectively; two neuroendocrine neoplasm; three cases of low‐differentiated adenocarcinoma, small cell carcinoma and thymoma, respectively; and four lymphoepithelioma‐like carcinoma. One case with three samples that the histologic diagnosis was small cell carcinoma, a significant CD45‐negative population of intermediate sized cells was identified by FCM in three groups. We added the cytokeratin marker to confirm the epithelial origin. The other 19 cases (55 samples) of non‐hematopoietic system diseases were negative by FCM studies. In these non‐hematopoietic system diseases, the concordance rates were 100% in both groups of CNB‐RT and CNB‐TCS but with a lower rate of 94.4% (17/18) in group FNA‐TCS. The discordant case in group FNA‐TCS was abnormal T‐cell by FCM analysis, whereas the histologic diagnosis was thymoma.

For DLBCL cases, 22 of 26 samples (84.6%) in group CNB‐RT, 20 of 26 samples (76.9%) in group CNB‐TCS and 21 of 26 samples (80.8%) in group FNA‐TCS were successfully identified by FCM studies. Overall, there were in total 15 of 78 DLBCL samples (19.2%) had a negative FCM diagnosis, probably due to those large neoplastic cells did not survive the harsh of tissue sampling and FCM processing.

Discordance between FCM and morphologic diagnosis was mainly identified in unclassified B‐cell lymphoma (B‐UCL) and T‐cell Lymphoma among three groups. Two cases (total six samples) of B‐UCL were both diagnosed as B‐cell lymphoma, unclassifiable, with features intermediate between DLBCL and CHL by morphology whereas the FCM results were all negative in three groups. This directly led to the concordant rate of B‐UCL in all three groups being zero. For T‐cell lymphoma, there was only one out of three samples (1/3, 33.3%) was concordant in groups CNB‐RT and CNB‐TCS, respectively; while one of two samples (1/2, 50%) was concordant in group FNA‐TCS. The concordant samples in three groups were derived from the same patient that was diagnosed as T‐cell prolymphocytic leukemia by morphology. The other two discordant samples were diagnosed as angioimmunoblastic T‐cell lymphoma (AITL) and NK/T‐cell lymphoma by morphology while the FCM results were all negative.

**Figure 1:**
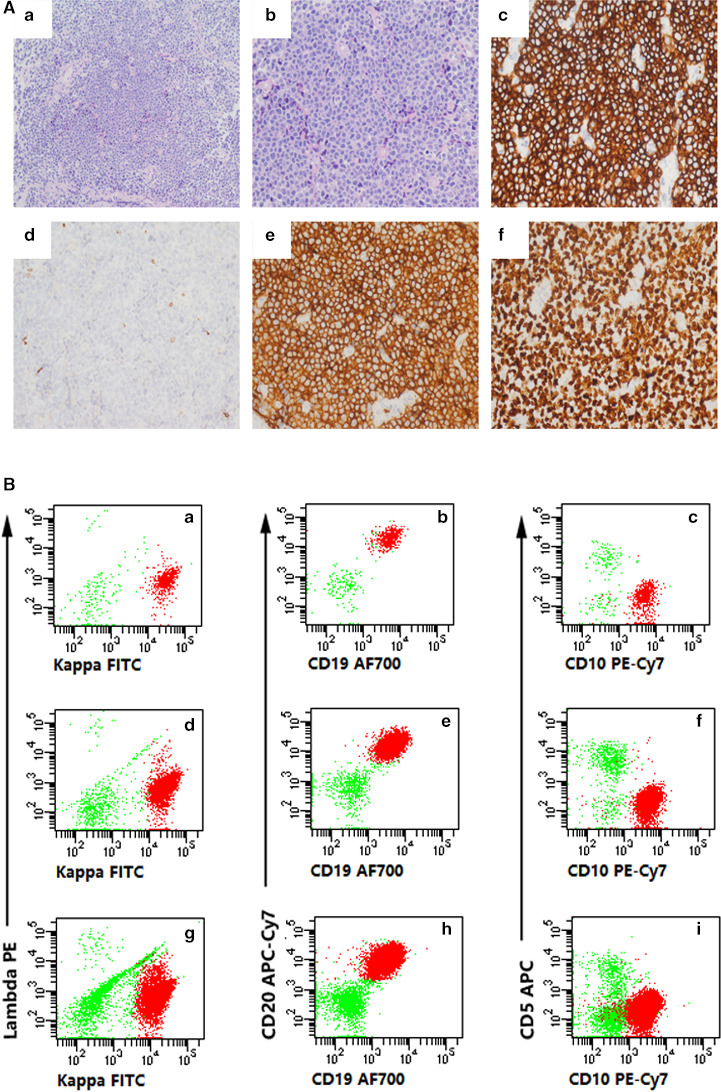
Cytological examination of the core needle biopsy (CNB) specimen and flow cytometric (FCM) evaluation obtained from retroperitoneal mass in a 53‐year‐old man with diagnosis as diffuse large B‐cell lymphoma (DLBCL), germinal centre B‐cell (GCB) subtype. A, Morphology and immunophenotype of retroperitoneal mass. (a) The biopsy shows diffuse infiltrate of lymphoid cells (H&E, ×200). (b) Higher magnification view shows uniform large neoplastic cells with prominent nucleoli (H&E, ×400). (c) All the neoplastic cells are strongly positive for CD19 (IHC×400), and (d) negative for CD3 (IHC×400). (e) Tumor cells are positive for CD10 (IHC×400). (f) The Ki67 proliferation index is >90% (IHC×400). B, Cell suspensions obtained by three different preparation methods, namely CNB‐RT (CNB rinsing technique), CNB‐TCS (CNB‐tissue cell suspension), and FNA‐TCS (fine needle aspiration‐tissue cell suspension), were detected by FCM. Figures (a) to (c), (d) to (e), and (g) to (i) represent the FCM results of groups CNB‐RT, CNB‐CTS, and FNA‐CTS, respectively. All lymphoid cells are shown in each group. T‐cells are highlighted in green. A clonal population of immunophenotypically abnormal B‐cells with Kappa light chain bright positive, CD19, CD20, and CD10 moderate positive, Lambda light chain and CD5 negative is highlighted in red. The clonal mature B‐cell population is detected in 78.10% (670/858 events), 85.12% (4364/5127 events), and 85.94% (13487/15693 events) of lymphocytes in groups CNB‐RT, CNB‐CTS, and FNA‐CTS, respectively

Herein, we presented a case of DLBCL, germinal centre B‐cell (GCB) subtype detected by morphology and FCM using three cell suspension preparation methods in Figure 1. The tissue specimens were obtained from a 53‐year‐old man with a retroperitoneal mass. Morphology evaluation revealed a diffuse pattern with a predominance of large cells in the CNB specimen. Immunohistochemical results showed that the tumor cells were B‐cells lineage with a high proliferation index (Ki67>90%). FCM results showed a population of immunophenotypically abnormal B‐cells with Kappa light chain bright positive, CD19, CD20, and CD10 moderate positive, Lambda light chain and CD5 negative in each group (highlighted in red). All lymphocytes were shown at 100%. Although the numbers of lymphocytes and abnormal B‐cells in the group CNB‐RT were the fewest in three groups, monoclonal mature B lymphocytes could still be detected.

### 3.3 Diagnostic sensitivity and specificity of the three cell suspension preparation methods

The values of sensitivity and concordant rate of each disease category were the same according to the definition of sensitivity. There was no significant difference in the diagnostic sensitivity of the three cell suspension preparation methods, although the value of group CNB‐RT was slightly higher. The specificities of groups CNB‐RT, CNB‐TCS, and FNA‐TCS were very high, reached 100%, 100%, and 98.8%, respectively (Table 2). There was no significant difference in specificity among three groups either. In group FNA‐TCS, one case that diagnosed as thymoma by morphology was misjudged to T‐cell lymphoma by FCM analysis. We could calculate the specificity of the T‐cell lymphoma to 98.8% (83/84) according to the definition of specificity.

## 4 DISCUSSION

In this study, we developed a rinsing technique (RT) using CNB cores to prepare the cell suspension for FCM analysis. We found that though the cell number of suspension in group CNB‐RT was the fewest among three groups and there was a significant difference (*p* < 0.001), it could yield sufficient cells for FCM immunophenotyping analysis. Furthermore, the concordant rate of which was gratifying and was not inferior to that of groups CNB‐TCS and FNA‐TCS (*p* = 0.819). FNA specimen usually mixed with blood due to the aspiration movement during sampling, furthermore, groups CNB‐RT and CNB‐TCS shared the same CNB tissue core. These two reasons could well explain why the groups CNB‐RT and CNB‐TCS had fewer cells than group FNA‐TCS. In addition, the number of QNS cases in the CNB‐RT was the same as that in the group CNB‐TCS (three cases), and less than that in the group FNA‐TCS (seven cases). In six of seven QNS cases in group FNA‐TCS, the corresponding cases in groups CNB‐RT and CNB‐TCS were able to obtain enough cells for FCM study (Table S1). This indicated that the CNB‐RT offered the superior quantity and quality of cell suspension for FCM and did not cause more artifacts and cell death that affect the results during process compared with the TCS technique.

To be more specific, for low‐grade B‐cell lymphoma, CHL, reactive, atypical/suspicious, and non‐hematopoietic system diseases, our study showed that the sensitivity (concordant rate) and specificity of group CNB‐RT were both 100%, which were better than or equal to that of groups CNB‐TCS and FNA‐TCS. In these types of diseases, not only the techniques of CNB‐TCS and FNA‐TCS, but also CNB‐RT strongly supported the final diagnosis.

For DLBCL, the sensitivity (concordant rate) of group CNB‐RT (84.6%) was lower than the above five diseases entities (100%). However, the specificity of group CNB‐RT was also 100%, indicating a high true negative rate. Some scholars conclude that in cases of DLBCL, the malignant morphologic features are typically so apparent that FCM analysis is often not necessary for a confident morphological diagnosis of lymphoma.^11^ Besides, FCM analysis of DLBCL would probably be falsely negative, mostly attributable to a low percentage of neoplastic cells seen in the insufficient FCM specimens. More fragile and less resistant to mechanical maneuvers such as vigorous aspiration are the causes to the destruction for the large‐cell lymphoma cells.^8^ One previous retrospective article about RT method suggested that more cases from deep‐seated sites and a higher percentage of DLBCL cases might result in a lower concordant rate.^21^ However, there were case selection biases which the patients and case numbers in the RT and TCS groups were discrepant. Our prospective trial was a parallel and double blind study, designed to make sure the same baseline among the three groups and the comparison would be more objective. In our study, after excluding the QNS samples, the proportions of DLBCL test samples in the three groups were similar, 28.9% (26/90) in both the groups CNB‐RT and CNB‐TCS, and 30.2% (26/86) in the group FNA‐TCS. Among all the DLBCL cases tested by FCM in the three groups, the deep‐seated lesion site accounted for the majority (78.6%, 22/28) and the superficial lesion site only accounted for 21.4% (6/28). However, all the 15 discordant samples of DLBCL in the three groups were deep‐site cases, which may be another reason for the higher discordant rate of DLBCL except the fragility of the large cells. Fibrosis is commonly present in the deeply seated body cavity lesions such as in mediastinal and retroperitoneal lymphomas.^8^ In our research, 13 of 15 discordant samples of DLBCL were easily detected with more fiber and extensive necrosis by the histological evaluation.

B‐UCL and T‐cell lymphoma were the two disease categories with low sensitivity (concordant rate). Two B‐UCL cases (both diagnosed as unclassifiable, with features intermediate between DLBCL and CHL) could not be detected by all the three methods which resulted in a concordant rate of 0%. However, the other seven disease categories had not been misdiagnosed as B‐UCL, thus, the specificities of all three groups in B‐UCL disease was 100%. Since grey zone lymphoma usually did not express surface immunoglobulins, if these large cells did not die during processing, we could see some large B‐cells with negative for both light chains, which were abnormal. Nevertheless, we could not observe these FCM results. We thought either the large cells died during the processing or unsuccessful sampling resulted in the discordance. Extensive necrosis or much less tissue was seen under the microscope in these two B‐UCL cases. For T‐cell lymphoma, the sensitivity (concordant rate) of group CNB‐RT (1/3; 33.3%) was not superior to group CNB‐TCS (1/3; 33.3%) and group FNA‐TCS (1/2; 50%). The specificity of CNB‐RT was 100%, which was equal to that of group CNB‐TCS and slightly better than that of group FNA‐TCS (98.8%). Although FCM analysis was helpful in identifying an abnormal T‐cell population, not all T‐cell lymphomas had distinct immunophenotypes. The inherent limitations and the sampling error could add the difficulty of FCM analysis for T‐cell lymphoma.

Whether the sampling is successful or not as well as the status of specimen directly affects the FCM results, either the RT method or the TCS method. We noted that almost all the QNS samples (12/13; 92.3%) for FCM analysis in three groups although the pathological examination gave a definite diagnosis; there were more necrosis, fibers, fat, or connective tissue in the background under the microscope. Therefore, it was comprehensible that the cell numbers of suspensions sampled from the same lesion site for FCM study would be insufficient. Furthermore, 24 of 28 (85.7%) discordant samples could also be observed with extensive necrosis and more fiber by the histologic detection, hence, the sampling errors could not be excluded in the discordant cases either.

In general, our results indicated that RT had a high sensitivity and specificity on assisting diagnosis for the cases with suspicious lymphoma, especially for the low‐grade B‐cell lymphoma, followed by DLBCL. The specificity of RT was superior to the sensitivity, which was the same as those reported by TCS method.^27^, ^28^ The FCM result by CNB‐RT is a powerful adjunct to the diagnosis of lymphoma. This prospective trial was a parallel and double‐blinded study with the same baseline among the three groups, which designed to make the comparison to be more objective. However, there were still some limitations in our study. First, the cases of low‐grade B‐cell lymphoma and DLBCL were not very many in each group; in addition, more cases are needed to evaluate the role of FCM analysis in B‐UCL and T‐cell lymphoma since the number in our study was too small for a definitive conclusion. Second, CNB‐RT could not avoid sampling bias; sampling nonviable tumor tissues such as necrosis, fibers, fat, or connective tissue in the lesion could also affect the FCM result using this technique. Finally, the FCM results of CNB‐RT were not embedded into the pathological diagnosis of lymphoma in this study, and that is what we need to research next.

## 5 CONCLUSION

RT is a simple, rapid, and effective way to preserve the maximum amount of tissue for lymphoma diagnosis and research while providing the suitable quantity and quality cell suspension for FCM detection as compared to the traditional TCS technique. RT can replace TCS technology, especially in cases with very few tissues. Therefore, we advocate the application of RT in the primary diagnostic procedure for patients with suspected lymphoma.

## CONFLICT OF INTEREST

The authors confirm that there are no conflicts of interest.

## AUTHOR CONTRIBUTIONS

Pei‐Dong Chi: Data analysis of FCM immunophenotyping, methodology, and writing‐original draft, data curation, and formal analysis. Yi‐Jun Liu: Treatment of tissue specimens and FCM immunophenotyping detection. Yu‐Hua Huang: Pathological evaluation of lymphoma. Ming‐Jie Mao: Statistics of data. Yu Wang: Resources. Zhi‐Ming Li: Resources, supervision, and writing‐review and editing. Jian Li: The sampling of tissue specimens, conceptualization, supervision, and writing‐review and editing.

## Supporting information

Table S1

## Data Availability

The authenticity of this article had been validated by uploading the key raw data onto the Research Data Deposit public platform (www. researchdata.org.cn), with the approval RDD number as RDDA2020001528.
