# Supplementary material for: Rinsing sampling of core needle biopsy for flow cytometric analysis: A favorable method for lymphoma diagnosis
Source: Cancer Med. 2020 Oct 17;9(24):9336–45. doi: 10.1002/cam4.3540 (PMC7774716; doi:10.1002/cam4.3540)
Supplement: Supplementary file 1 — Table S1 [file CAM4-9-9336-s001.docx]

Table S1. Distribution of the Sequence Number of Quantity Not Sufficient Cases for FCM Analysis in Three Cell Suspension Preparation Methods According to the Morphological Diagnosis Category

| Morphological Diagnosis^d^ | CNB-RT^a^ |  | CNB-TCS^b^ |  | FNA-TCS^c^ |
| --- | --- | --- | --- | --- | --- |
|  | n=3 |  | n=3 |  | n=7 |
| Diffuse large B-cell lymphoma | 22^nd^/52^nd^ |  | 52^nd^/89^th^ |  | 64^th^/79^th^ |
| T-cell lymphoma | 59^th^ |  | 59^th^ |  | 59^th^/88^th^ |
| Reactive^e^ |  |  |  |  | 26^th^ |
| Non-hematopoietic system diseases^f^ |  |  |  |  | 63^rd^/68^th^ |

^a^CNB-RT, core needle biopsy-rinsing technique.

^b^CNB-TCS, core needle biopsy-tissue cell suspension.

^c^FNA-TCS, fine needle aspiration-tissue cell suspension.

^d^Diagnosis category referred to the overall final histologic diagnosis.

^e^Reactive included the negative or granulomatous lymphadenitis cases diagnosed by histology.

^f^Non-hematopoietic system diseases included gastrointestinal stromal tumor, lymphoepithelioma-like carcinoma, neuroendocrine neoplasm, low-differentiated adenocarcinoma, low-differentiated squamous cell carcinoma, thymoma, small cell carcinoma, fibroma, lymphopapillary cystadenoma and neurinoma.
